# Analysis of UV Curing Strategy on Reaction Heat Control and Part Accuracy for Additive Manufacturing

**DOI:** 10.3390/polym14040759

**Published:** 2022-02-15

**Authors:** Fengze Jiang, Dietmar Drummer

**Affiliations:** Institute of Polymer Technology (LKT), Friedrich-Alexander-University Erlangen-Nuremberg, Am Weichselgarten 10, 91058 Erlangen, Germany; dietmar.drummer@fau.de

**Keywords:** photopolymerization, curing strategy, reaction heat, shrinkage and warpage, additive manufacturing

## Abstract

In this research, the relationship between the curing strategies and geometrical accuracy of parts under UV light was investigated. An IR camera was utilized to monitor the process using different combinations of photosensitive resin and curing strategies. The influences of curing strategies on different material compositions were studied with single-factor analysis. With the different exposure frequencies of the UV light, the peak temperature was adjusted to avoid overheating. The three-dimensional geometry of casting tensile bars was measured to investigate the shrinkage and warpage during the curing process. Different material compositions were also selected to study the effects of the maximum temperature on the shrinkage of the parts. The findings of this work show that, with the same amount of energy input, a more fragmented exposure allows for a more controllable max temperature, while one-time exposure leads to a high temperature during the process. With the decrease of the released heat from the reaction, the shrinkage of the casting part has a slightly increasing tendency. Moreover, the warpage of the parts decreased drastically with the decrease of temperature. The addition of fillers enhances the control over temperature and increases the geometrical accuracy.

## 1. Introduction

UV curing additive manufacturing is one of the most important branches of the additive manufacturing system. With the ever increasing expansion of the UV curing system, especially for desktop stereolithography (SLA) and digital light processing (DLP) printers, UV curing-based additive manufacturing elicits much attention from both academia and industries [1]. After the development of the fast digital light synthesis (DLS) printer, UV curing printed parts have progressed even more from prototyping to being directly used industrialized parts along with those manufactured using traditional manufacturing methods [2,3,4]. Moreover, since the curing requires less energy and a faster reaction rate, the high efficiency and environmentally friendly characteristics are of great importance [5].

Although UV curing-based additive manufacturing has benefits such as a high resolution, smooth surface quality, and relatively fast printing, there are still several limitations that affect the printing speed [6,7]. One of the most important factors is the reaction heat. For free radical reactions, a massive amount of heat is released during curing because of the breakdown of the carbon double bond [8,9]. With the development of personalized applications, such as tooth printing, the minimization of the resin amount to avoid the production of waste and reduce the cost is greatly desirable [10]. The lesser the amount of resin, the harder it is to exchange the released heat with the surrounding liquid based on a lower heat capacity. Since the regular thermoset resin is of a low heat conductivity, the accumulated heat is problematic, including an inadequate surface quality, insufficient mechanical performance, and low geometry accuracy [11,12,13]. There are several solutions available on the market to avoid these issues, but they have rarely been systematically discussed. The initial temperature of resin on the mechanical properties of parts has been discussed, which could reach a better surface quality but did not clearly change the mechanical properties [8]. Researchers have tried different methods to solve this issue: the classic path is lifting the printing platform to let parts cool in the air and using a stirring bar to remix the resin tank to help heat distribution; the surface exposure method using micro shaking of the platform through a vacuum effect promotes the resin heat exchange between the printing area and non-printing area; and, from the chemical side, using a thinner layer or smaller exposure area to limit the heat release in the unit time can partially solve the problem. However, most of these solutions result in a lower printing efficiency. To maximize the printing speed with a large printing area, there is a dynamic cooling method that uses laminar circulating cooling oil which is running under the printing area to cool down the entire resin tank [6], but it requires extra structure and a circulation system. Composite resin, with different fillers inside, will also affect the absorption and reflection of the UV light; however, the majority of the research focuses more on the final part properties and the double bond conversion rate [14,15].

Shrinkage and warpage is another concern with regard to the free radical cured resin, the average shrinkage reaching between 5 and 20% depending on the selection of oligomers and monomers [16,17]. During the printing process, once a certain level of shrinkage and warpage are achieved, the distortion of the focal plane will increase the number of errors on the Z-axis that eventually deteriorate the integrity of the products. The delamination between layers generated from the shrinkage and warpage is one of the major drawbacks of UV curing additive manufacturing [18,19]. The origins of the problem can be classified into several major factors: chemical reaction, residual stress, cooling time, etc. [10,20,21]. The heat expansion and linear shrinkage after curing were discussed and a model was built [22]. The solution is to lower the intensity and slow down the average reaction rate, and also change to an optimized resin that has a lower shrinkage rate. The fillers could help decrease the shrinkage and warpage; however, information on the changing curing strategies of the filler resin is still missing [23].

In summary, the effect of the reaction heat released on the part during the process has not been fully discussed yet since the temperature increases the reactivity of the free radical reaction while at the same time dramatically decreasing the viscosity of the resin and heat expansion. The main focus of this research is to observe the in situ heat released during the printing process and compare the properties of the obtained final parts, including the part shrinkage and the warpage rate. These results could help investigate and optimize the processing parameters and promote the final properties of the part with a relatively small effect on the printing efficiency. Thus, it is essential to unearth the effect of the reaction heat during the curing process to improve the final printing accuracy.

In this paper, we first systematically analyze the effect of the curing strategies on the maximum local reaction temperature with the customized resin using an IR camera. The maximum temperature and the effects of different temperatures on the part shrinkage and warpage after the parts were cured are discussed. Moreover, two different shapes of fillers were added into the resin to investigate the basic effects of composite materials’ shrinkage and warpage compared with the non-filler resin.

## 2. Methods

### 2.1. Preparation of Hybrid Resin

The matrix of the resin was prepared using aromatic urethane acrylate (3-isocyanatomethyl-3,5,5-trimethylcyclohexyl isocyanate, Photomer 6628) as the oligomer, HDDA (Photomer 4017) as the reaction diluent, and Photoinitiator 1173 (Omnirad 1173). UV resin was purchased from IGM Resins, Netherlands.

Extra agent and fillers were also added to the resin based on the different demands of the prepared resins. Fumed silica (Aerosil 200, Evonik, Germany), with an average particle size of 12 nm, was used as the viscosity and thixotropic agent, while glass spheres and short glass fibers were implemented during the casting process. The glass spheres had a particle size between 30 and 50 µm, and the short glass fibers had an average length of 200 µm, as shown in Table 1 and Table 2. The filler amount was set at 5% based on the testing limitation and reference [24].

The resins and fillers were mixed by the centrifugal mixer (ARE-310, Thinky Inc., Laguna Hills, CA, USA) with 2000 rpm for 6 min to disperse all the components uniformly. After mixing, the mixed resin was gently filled into a syringe to prevent bubbles and was allowed to cool to room temperature. The material compositions are shown in Table 3.

### 2.2. Casting Mold

**Figure 1:**
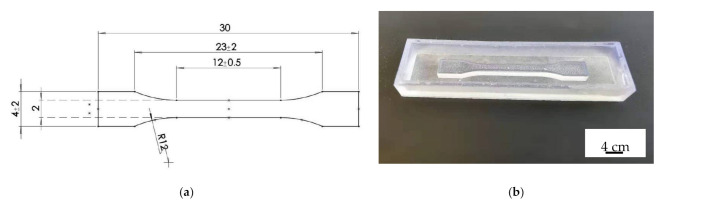
The casting mold of (**a**) the 1BB type geometry of tensile bar and (**b**) the male casting mold made by SLA.

The standard dog bone samples were prepared in accordance with the ISO 20753, standard type 1BB. An SLA printer was initially adopted for the male casting mold followed by the replication on PDMS to the female mold which is shown in Figure 1.

### 2.3. Temperature Observation

The UV oven (AMP Technica AG, Germany) was equipped with three levels of light intensities. In order to assure the exposure of an equivalent amount of light energy to all samples together with the control of the reaction rate using the light intensities, the lowest level of light intensity (10 mW/cm^2^) was selected. The sample was positioned in the middle of the two UV led lamps, for which the position was labeled to ensure all samples were placed in identical locations.

**Figure 2:**
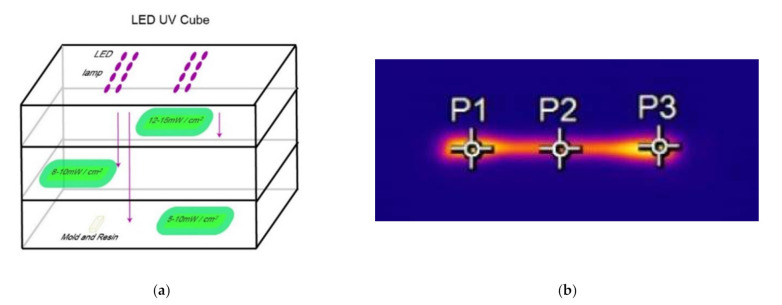
(**a**) Inner structure of UV oven and (**b**) sampling positions of IR camera.

The IR instrument was placed at the corner of the oven with a proper angle to monitor the curing process of the tensile bar by three points (Figure 2b) which divided the bar into three parts. Moreover, the average temperature during the reaction was recorded for the calculation of heat released during the mold casting.

### 2.4. Curing Strategies

**Figure 3:**
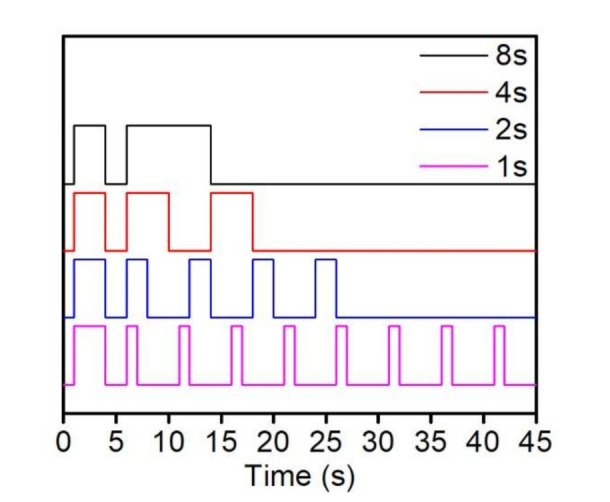
The curing strategies by control UV light.

During the previous research, the material had about a 3 s initiation time to generate sufficient free radicals to eliminate the oxygen and initiate the curing [25]. In comparison with the commercial printer, the ratio between the light being on and off is about 3:7; thus, we designed several groups of curing strategies to determine the effect on the average temperature of the resin in the mold. Intensive pre-testing of the suitable curing time was conducted to investigate the position where the temperature remains unchanged while oxygen has been consumed. Due to the massive heat release during curing, we decided to disjointedly expose the tensile bars to UV light with various fragmented UV exposure times, while keeping the total UV exposure time identical (8 s). The fragmented UV exposure time were 4 s, 2 s, and 1 s, which requires the repetitive exposure to UV light 2, 4, and 8 times, respectively, with 4 s time intervals, as shown in Figure 3 [25]. The detail experiment groups are shown in Table 4. The curing time was set up on the UV oven control panel and the time interval was fixed to 4 s.

### 2.5. Shrinkage and Warpage

**Figure 4:**
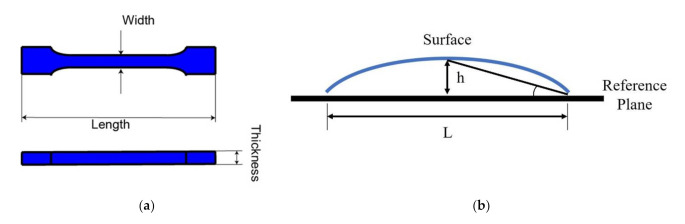
(**a**) Shrinkage measurement position and (**b**) warpage measurement angle.

The pronounced shrinkage, together with the large heat release during UV curing, affects the stress distribution of the residues, which leads to the strong warpage that greatly hinders the application of UV curing resin. Thus, it is of importance to investigate the heat released from the curing, which was identified by the tensile bar geometry and warpage angle. For the tensile bar, the width, length, and thickness were measured three times for each bar, and the warpage angle was measured with the protractor. The shrinkage measurements were taken with the width and the length of the tensile bar flattened on the table as shown in Figure 4a, and the warpage was measured using the angle between the flat surface and the highest position as shown in Figure 4b.

## 3. Results and Discussion

### 3.1. Effects of Different Curing Strategies and Fumed Silica Ratio on Reaction Heat Control

Two types of resin with different amounts of fumed silica were measured, which was used to adjust the initial viscosity based on the high surface area of the nanoparticles. However, to investigate the resin at a certain viscosity, the effects of the fumed silica on temperature need to be clarified.

**Figure 5:**
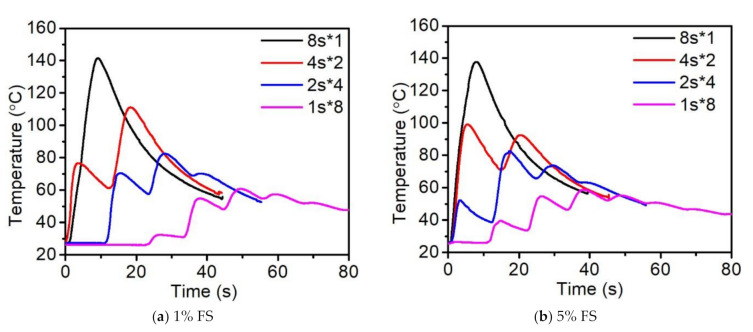
Effects of fumed silica amount and curing strategies on the reaction heat temperature.

Although the peak temperature reveals a minute difference, the increase of temperature was significantly altered in the presence of the fumed silica, which indicates that the nanoscale silica particles may reflect the UV light to the surroundings, enhancing the curing. Comparing Figure 5a and Figure 5b, the first peak of the temperature was reached in a shorter period of time with a higher value when 5% FS was added, except for at the 8 s exposure time, which may be due to the attainment of the highest conversion. For example, at a 4 s curing time, the temperature of 5% FS was 20 °C higher than that of 1%, while, at a 2 s curing time, the 1% FS curve revealed no peak while the appearance of a peak in 5% FS was detected.

Moreover, it was found that the curing strategies greatly affect the value of peak temperature; the was higher the frequency of the cooling intervals, the lower was the peak temperature. The above-mentioned conclusion was important for the preparation of the materials because, for low flash point materials, a lower peak temperature facilitates the reduction of heat residual stress, enabling the uniform distribution of temperature.

In general, the addition of 5% fumed silica did not drastically decrease the temperature during the curing process, and since it was utilized as the thixotropy agent for the resin, the excess addition of fumed silica decreased the flowability of the resin. However, it should be noted that the increase of the fumed silica content up to 5% results in a faster curing with a more uniform distribution [15,26].

### 3.2. Effects of Different Curing Strategies and Filler Types on Reaction Heat Control

**Figure 6:**
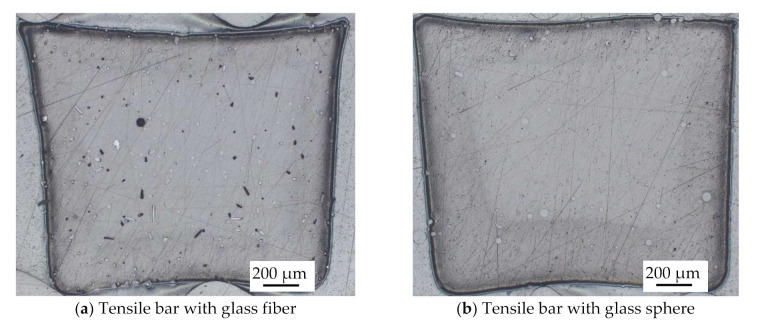
Polarizing microscope image of cured composite resin cross-section area.

The polarized microscope image in Figure 6 shows the distribution of fillers in the middle region of the tensile bar. Both the glass spheres and glass fibers were uniformly distributed in the bar. Moreover, in terms of the orientation of the glass fiber, as shown in Figure 6a, it was perpendicular to the paper, where the transparent holes indicate the fiber remained in place while the dark holes indicate the absence of the fiber.

**Figure 7:**
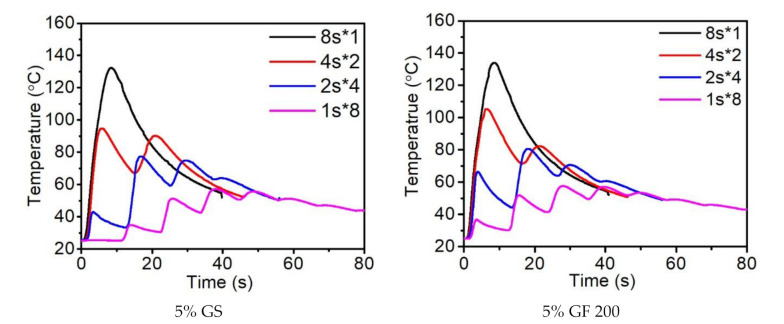
Effects of filler type and curing strategies on the reaction heat temperature.

In Figure 7, two types of fillers—glass spheres and short glass fibers—were added into the resin to study the effects of different fillers on heat control during curing. From the above-mentioned observations of fumed silica, the fillers were uniformly distributed in the resins and could maintain the dispersion for at least a month.

In the case of an 8 s curing time, the difference between the first peak temperatures of the two fillers was insignificant; however, higher heat releases within a shorter curing time were observed for both fillers. The results may due to the fact that under a relatively long light exposure time (8 s), the impact of fillers on the curing process is negligible.

With a 4 s curing time, the glass spheres as filler demonstrated a milder heat release at the first peak (93 °C) in comparison with that of the glass fibers (105 °C). With a 2 s curing time, the glass fibers as filler revealed a higher first peak of 66 °C compared with the glass spheres’ peak of 43 °C, which indicates the higher curing degree of the resin. The results may due to the inherent anisotropic structure of the glass fiber, which outcompetes the isotropic structure of the glass sphere in regard to the facilitation of curing since the oriented microstructure of the glass fiber reflects the light between fillers more efficiently.

**Figure 8:**
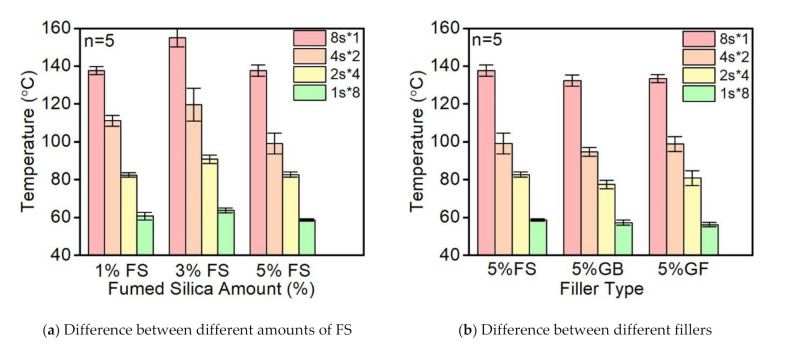
Comparison of the reaction temperatures using different curing strategies.

Figure 8 presents the comparison of the peak temperatures during the curing process under different conditions. Figure 8a illustrates that with the increase of the loadings of fumed silica nanoparticles from 1% FS, the maximum reaction temperature first increased at the 3% FS loadings followed by a decrease down to its original level at the 5% FS loadings. Thus, the optimal loading of fumed silica nanoparticles that promotes the absorption and reflection of the UV light is 3% FS. Moreover, once the exposure time decreased, the decline of the average reaction temperature was significant even under identical total energy input. Regardless of the exposure time, the 3% FS loadings reveal an increase in reaction temperature and the discrepancy between 8 s and 1 s was as large as 60 °C, which decreases the thermal stress during the cooling stage and increases the printing accuracy.

The comparison between different types of filler was also investigated. As shown in Figure 8b, the reaction heat was slightly reduced regardless of the type of filler, which further decreased the maximum reaction temperature during curing. The effects of fillers on curing depended on the shape of the fillers.; the fumed silica nanoparticles revealed the least impact while the glass spheres decreased the reaction temperature to a higher degree compared to the glass fibers. In addition to the reaction temperature, the shape of fillers may also affect the UV absorption and reflection during light exposure; however, the difference of temperature was insignificant due to the low loading of fillers (5%).

### 3.3. Effects of Curing Strategies on Parts Volume Shrinkage

UV-initiated radical curing is known for its shrinkage during the printing process, which drastically decreases the printing accuracy of parts and increases the printing difficulties. Thus, we tend to measure the shrinkage and warpage of the parts under different curing conditions with various curing strategies.

**Figure 9:**
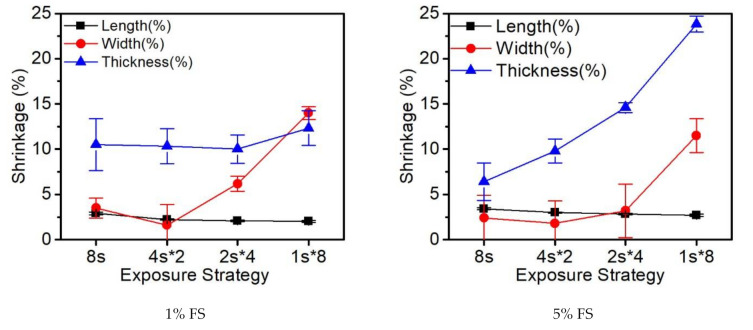
The shrinkage of tensile bar with different amounts of FS.

As shown in Figure 9, the effect of fumed silica loadings on the shrinkage of parts in three dimensions was measured. The fumed silica was initially regarded as a thixotropic agent and the suitable loading range for direct writing printing was selected from 1% to 5%. From the graph, we noticed that the increase of fume silica loadings barely impacted the length and width of the tensile bar; however, with regard to the thickness, the increase of the fumed silica content greatly increased the shrinkage. Moreover, the decrease of the fragmented exposure time from 8 s to 4 s, 2 s, and 1 s shows pronounced increases for the shrinkage of width and thickness, which may due to the fast curing of the tensile bar surface under a relatively long UV exposure time (8 s) that prevents the penetration of light into the inner layer.

**Figure 10:**
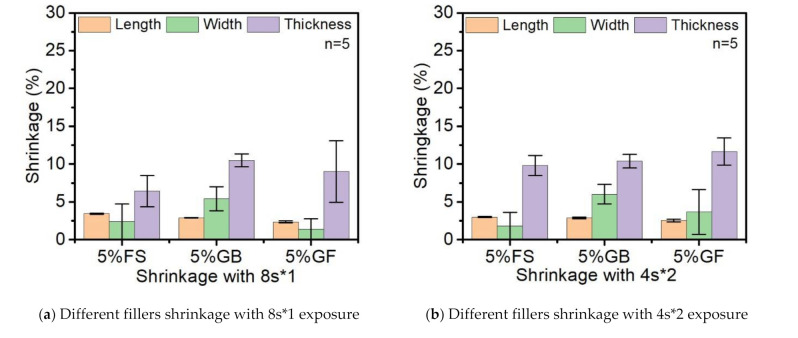

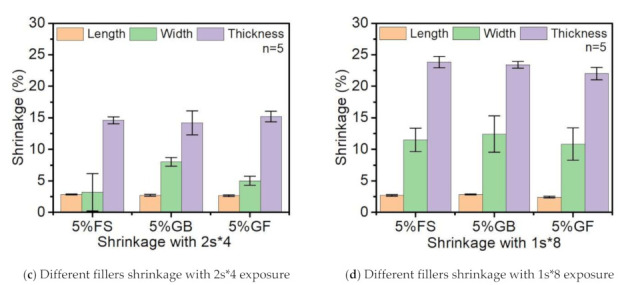
The shrinkage of tensile bar under four curing strategies with different fillers.

In Figure 10, the comparison between different materials and curing strategies on the shrinkage of tensile bars in three dimensions is shown. In general, the shrinkage in length was as low as 3%, which is insignificant in terms of free radical curing. Comparatively, the shrinkage in width is obvious for both the glass fibers and glass spheres and is more distinct in the latter case. In terms of thickness, due to the casting method, one side of the surface is exposed to the atmosphere, which leads to significant differences in comparison with the other two directions in shrinkage. As a result, the shrinkage in thickness increased, varying from 14% to 23% depending on the different curing strategies and materials. Conclusively, with the increase of fragmented exposure time, barely any changes were detected in length, the width increased gradually, and the discrepancy in thickness was the most significant, which may have been due to the oxygen inhibition.

### 3.4. Effects of Curing Strategies on Parts Warpage

**Figure 11:**
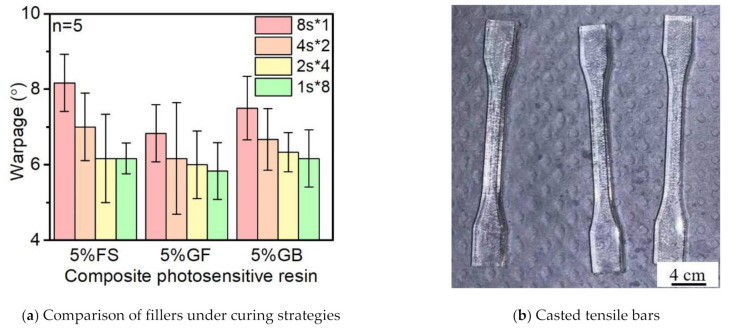
The warpage of the tensile bars with different fillers.

From the results of the warpage studies in Figure 11, the addition of glass spheres and glass fibers drastically decreased the warpage degree, while the glass fibers resulted in a higher degree of decreasing which may have been due to the shape and orientation of the glass fibers that enhanced the flexural strength along the axial direction. By following the direction of the mold, the glass fibers were oriented and parallel to the surface after casting.

The casting direction was extruded along with the mold, so the glass fibers were orientated by the liquid flow and kept parallel to the surface. At this position, the floating glass fibers can reinforce the tensile bar structure to resist the deformation and residual stress.

In addition, with the decrease of the fragmented exposure time, the warpage angle decreased at the same time, which means the warpage maintains the flat surface that allows a second layer of deposition during the additive manufacturing process.

## 4. Conclusions and Outlook

In this paper, the UV curing for casting parts was investigated to study the maximum reaction heat, shrinkage, and warpage under different curing strategies and types of fillers. The differences in curing strategies with identical total exposure times were evaluated to understand the effects on temperature control. Moreover, the effects of the type of filler were also tested to understand how the shape of fillers affects the heat release. The results show that with the increase of the loading of fumed silica, the reaction heat first increased and then decreased; however, the shrink in width and thickness was increased, especially in the fragmented exposure time of 1 s. On top of that, the addition of fillers led to a slight decrease in the maximum reaction heat, and it should be noted that the shortening of the fragmented exposure time, while keeping the total exposure time identical (8 s), accounted for the drastic decrease of the maximum reaction temperature. In light of the types of fillers, the addition of short fibers decreased the total shrinkage more than the glass spheres based on the anisotropic shape that changes the light path. Moreover, both the decreases of fragmented exposure time and the addition of fillers decreased the warpage of the parts, and the glass fibers showed a higher warpage resistance in comparison with the glass spheres.

The results of this paper provide guidance for material design and development for the UV curing resin based on different applications in terms of the model geometric accuracy. The studies of the two types of fillers on shrinkage and warpage also provide new routes for composite resins for functional printing purposes.

## Figures and Tables

**Table 1:** Milled glass fiber.

Filler	Glass Fiber	SEM
Brand	Taishan EMG-200	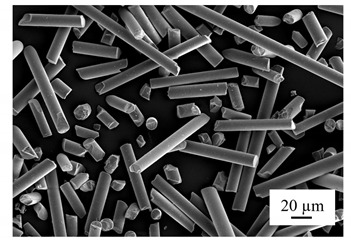
Type of glass	E-glass	
Sizing agent	None	
Filament diameter	≤38 µm	
Average length	200 µm	
Moisture	≤0.1	
Loss on ignition	≤0.2	

**Table 2:** Glass sphere.

Filler	Glass Sphere	SEM	Size Distribution
Brand	Spheriglass 3000	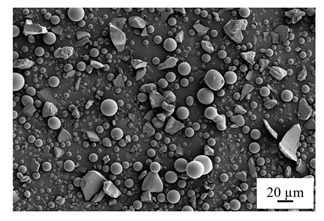	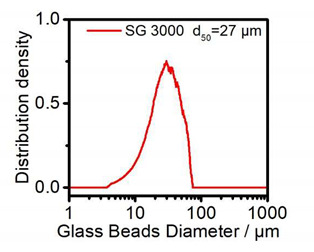
Type of glass	E-glass		
Particle size	30–50		
Bulk density	1.44 g/cm^3^		

**Table 3:** The composition of the test material (weight percentage).

	Oligomer	Monomer	Photoinitiators	Fumed Silica	Glass Sphere	Short Glass Fiber
1	58%	38%	3%	1%	-	-
2	57%	37%	3%	3%	-	-
3	56%	36%	3%	5%	-	-
4	54%	35%	3%	3%	5%	-
5	53%	34%	3%	5%	5%	-
6	53%	34%	3%	5%	-	5%

**Table 4:** The curing strategies.

Group	Exposure Time (s)								
1	3	8	-	-	-	-	-	-	-
2	3	4	4	-	-	-	-	-	-
3	3	2	2	2	2	-	-	-	-
4	3	1	1	1	1	1	1	1	1

## Data Availability

Not applicable.
